# Favorite Prescriptions of Distinguished Practitioners

**Published:** 1889-10

**Authors:** 


FAVORITE PRESCRIPTIONS OF DISTINGUISHED PRACTITIONERS, with note on Treatment, compiled from the writings and unpublished records of Drs. Fordyce Barker, R. Bartholow, S. D. Gross, Austin Flint, A. Clark, A. L. Loomis, F. J. Bumstead, T. G. Thomas, H. C. Wood, Wm. Goodell, J. M. Fothergill, N. S. Davis, J. M. DaCosta, J. Solis Cohen, J. L.

Smith, Brown-Sequard, etc. By B. W. Palmer, A. M., M. D. Published by E. B. Treat, New York, 1888.

This book is well bound, and contains 250 pages. To the physician who does not possess the tact of extemporising a prescription, this compilation ought to be valuable. It contains a great many very choice prescriptions and suggestions. B.

